# The subjective experiences of patients recovering from delirium in acute geriatric care: An analysis of quantitative and qualitative interview data

**DOI:** 10.1186/s12877-026-06992-z

**Published:** 2026-01-23

**Authors:** Svenja Tietgen, Isabell Behnen, Jessica Koschate-Storm, Milena von Kutzleben, Steffi Wiards, Tania Zieschang, Brooke C. Schneider, Alexander Rösler

**Affiliations:** 1 Department of Geriatrics, General Hospital Bremerhaven Reinkenheide gGmbH, Postbrookstraße 103, 27574 Bremerhaven, Germany; 2 Geriatric Medicine, Department for Health Services Research, Faculty VI Medicine and Health Science, Carl von Ossietzky University Oldenburg, Ammerländer Heerstraße 140, 26129 Oldenburg, Germany; 3 Division of Prevention and Rehabilitation Research, Department for Health Services Research, Faculty VI Medicine and Health Science, Carl von Ossietzky University Oldenburg, Ammerländer Heerstraße 140, 26129 Oldenburg, Germany; 4 Department of Geriatrics, General Hospital Lüneburg GmbH, Bögelstraße 1, 21339 Lüneburg, Germany; 5 Department of Psychiatry and Psychotherapy, University Medical Center Hamburg-Eppendorf, Martinistraße 52, 20246 Hamburg, Germany; 6 Department of Geriatrics, Agaplesion Bethesda Krankenhaus Bergedorf GmbH, Glindersweg 80, 21029 Hamburg, Germany

**Keywords:** Older people, Hospital, Anxiety, Fear, Memories, Hallucinations

## Abstract

**Background:**

Delirium occurs in about one fourth of hospitalized older adults and is associated with multiple adverse outcomes. Despite efforts to improve the prevention and treatment of delirium, it remains a burden for patients, their relatives, and healthcare professionals. Currently, little is known about how people experience delirium episodes.

**Methods:**

A sample of 77 inpatients who had been recently treated for delirium were recruited from acute geriatric care units at four hospitals. A semi-structured interview was conducted to explore memories and feelings associated with the delirium episode. The interview included both, dichotomous answers (yes/no) as well as the possibility to provide in-depth comments. Quantitative data were analysed using descriptive statistics. Qualitative data were analysed with content analysis. After three months, a follow-up interview was conducted with a subgroup (*n* = 29) via telephone.

**Results:**

A majority (*n* = 66, 85.7%) of participants could recall the delirium episode. The main emotion reported by patients related to the previous delirium episode were anxiety and/or fear (67.5%, *n* = 52). Visual hallucinations and/or dream-like experiences were recalled by nearly 60% (*n* = 46). Approximately one-fourth (*n* = 21) reported that they felt fully recovered from the episode, although another 25% (*n* = 21) reported lasting symptoms. Three months after the initial interview, ten participants reported persistent negative feelings associated with the episode of delirium. Conversations with staff and family members were found to be the most helpful intervention during and after the delirium. Likewise, guidance on orientation was found to be helpful during delirium.

**Conclusions:**

Anxiety and/or fear were the most common feelings reported after recovering from delirium. Negative memories and anxiety about cognitive decline were prevalent among those who failed to cope with delirium. Opportunities to talk about delirium and to provide reassurance to those affected should be integrated in usual care.

**Trial registration:**

https//www.drks.de/on07/01/2022 (DRKS00024078).

**Supplementary Information:**

The online version contains supplementary material available at 10.1186/s12877-026-06992-z.

## Background

Delirium in geriatric medicine occurs with a prevalence of approximately 25% in hospitalized patients and is associated with poor short- and long-term outcomes [1]. In the USA, the estimated number of cases of delirium in individuals aged 65 and above is about 2.6 million per year, with a significant proportion of cases remaining undiagnosed. Research has focused on multicomponent interventions, including education of staff and improving the treatment of risk factors and care during delirium [2, 3]. Whereas multicomponent non-pharmacological interventions for the prevention of delirium have been found to be effective, evidence for the treatment of delirium itself is still limited [3, 4].

Due to its high relevance, “emotional stress” has been incorporated into the core outcome set for delirium intervention studies [5], highlighting its importance for patients, relatives, and care professionals. Recognizing such stress is important as it may be a “lasting scar” of experiencing a delirium. Prompt and effective intervention for delirium-related distress is therefore of great importance.

To improve professional care, better understanding of patients` experience during and after an episode of delirium is essential. Few trials examining patients’ lived experiences of delirium have been published. Moreover, the published work to date has utilized small sample sizes (*n* < 15) or was conducted in intensive care units [6–8].

In this multicenter study we sought to explore whether patients in acute geriatric care remember experiencing an episode of delirium and, if so, could report which feelings were most prevalent during the episode, which memories were the most distressing, and which actions of staff or family were felt to be the most helpful.

## Methods

### Study design

In this multicenter study inpatients were recruited who had a diagnosis of delirium during their hospital stay at one of four sites in northern Germany (Univ. Ol, Hosp. B, Hosp. Bhv, and Hosp. L). All sites included patients from geriatric departments; at two sites, participants were additionally recruited from the Center of Geriatric Traumatology (Univ. Ol, Hosp. Bhv). The hospitals had implemented different delirium prevention-bundles. For further information please see additional file 2. Participants were recruited sequentially throughout the study period.

The study was approved by the Ethics Committee of the University of Oldenburg and was conducted in accordance with the Declaration of Helsinki in its current version. The study was registered at https://www.drks.de/on07/01/2022 (DRKS00024078). All participants provided written informed consent.

### Study sample

During the recruitment period from October 2020 to February 2023, a total of 77 participants at four sites were included (*n* = 25 Univ. Ol, *n* = 16 Hosp. B, *n* = 17 Hosp. Bhv, and *n* = 19 Hosp. L). None of the patients approached denied participation or withdrew the informed consent. Patients who developed delirium during their hospital stay were identified by an experienced physician or nursing staff, according to at least two of the following criteria: acute change in mental status, difficulty in focusing attention, disorganised thinking and disturbed or fluctuating alertness. Additionally, 10 patients were identified by Rapid Clinical Test for Delirium (4 AT) or Confusion Assessment Method (CAM). The resolution of the episode of delirium was defined by the presence of both a score in the “intact” range on a validated delirium test instrument (4 AT or CAM or CAM-ICU) in all cases, as well as clinical agreement by physicians and nurses. Following the resolution of the delirium, patients were informed about the study and recruited after giving written informed consent. The semi-structured interview was conducted only after consent had been given. At all sites, the interviews were conducted in person. At one site, (Univ. Ol.) these interviews were recorded. At two sites a follow-up via phone call after 12 weeks was performed (Univ. Ol, Hosp. Bhv). Inclusion criteria were met for patients undergoing geriatric treatment who were admitted with delirium or developed delirium during their hospital stay. Patients who recovered from delirium during the stay were included. Prior to the interview, the clinically experienced interviewer confirmed that the participant fully understood the purpose of the study as well as the interview questions. Exclusion criteria encompassed previously known severe dementia, as well as the presence of current or previous psychotic psychotic illnesses, mainly schizophrenia, schizoaffective disorders and mood disorders with psychotic features.

### Data collection and measurements

After recovering from delirium, patients completed a semi-structured interview with a trained interviewer. Due to the lack of established interview-tools for a post-delirium status, an interview guide was developed within the research group two months before the recruitment period started. This interview was not validated prior to use in the study. The senior author (AR) proposed a questionnaire consisting of eleven “yes/no” questions, which was discussed and revised in two team meetings. In addition to dichotomous responses, participants were encouraged to provide more detailed answers, which were noted by the interviewer during the interview. The interviewers sought to record as much information as possible and interviewees were asked follow-up questions as necessary to improve the richness of the responses. The questions focused on the episode of delirium, asking about anxiety and hallucinations, memories, experience of distress, perceived duration of the delirium, and the fear of experiencing a delirium in the future. Data collection and interviews were performed by a physician (Univ. Ol), geriatrician (Hosp. B and Hosp. Bhv.) or psychologist (Hosp. L). The English version of the interview guide is provided in the supplementary (additional file 1).

Patient characteristics were taken from the patient’s medical record (age, sex, body weight and height, sensory deficits, use of hearing aids, level of care, medication before, during, and after delirium, results of blood tests, primary diagnosis). Only the data deemed directly relevant to the research question is reported in this article. Cognitive function was screened using the Mini Mental State Exam (MMSE) as part of the standard geriatric assessment at admission. The score was obtained from patients’ medical record. Delirium length and severity was assessed by 4 AT, CAM, CAM-ICU, and from the daily documentation routinely conducted by nurses, and physicians. Causes and courses for the delirium were taken from patients’ records. Delirium was classified as hyperactive (predominantly increased motor activity and heightened alertness), hypoactive (mostly reduced motor activity and responsiveness), or mixed (fluctuations between these states). Postoperative delirium was defined if symptoms occurred within 0–3 days after surgery; delirium was attributed to infection if clinical and laboratory signs of infection were present. In a subgroup (*n* = 29), a follow-up telephone interview was conducted three months after the initial interview to examine potential changes in patients’ perception of the delirium, and health status.

### Data analysis

Quantitative data, obtained from the yes or no questions in the interview guide were analysed using descriptive statistics.

The qualitative data obtained from the patient’s open-ended responses were analyzed with qualitative content analysis [9]. The written narratives were reviewed and inductively coded by one researcher (ST). The results were discussed within the study team (JK-S, IB, MvK) in two rounds to validate the coding process and to identify overarching themes. Three relevant themes of patient’s experience could be identified: (1) emotions during delirium; (2) hallucinations and dream-like experiences, and (3) changes in feelings and memories over time/ coping.

## Results

Participants were, on average, 82.3 years of age (range 62–98) and 52% were female. The average MMSE score was 25.3 points (range 17–30). The mean body mass index was 25.9 (range 17.3–37.5). Visual impairment was present in 58.4%, hearing impairment in 28.6% of all participants. Patient characteristics are shown in Table 1.

**Table 1:** Patient characteristics

Age, mean (range)	82.3 (62–98)
BMI, mean (range)	25.9 (17.3–37.5)^a^
MMSE, mean (range)	25.3 (17–30)^b^
Length of hospital stay, mean (range)	24.5 (7–63)
Level of care at admission	
• Level 0 (limited daily living skills), n (%)	1 (1.3)^c^
• Level 1 (mild impairment of independence), n (%)	2 (2.6)^c^
• Level 2 (moderate impairment of independence), n (%)	23 (29.9)^c^
• Level 3 (severe impairment of independence), n (%)	11 (14.3)^c^
• None, n (%)	36 (46.8)^c^
Social situation before admission	
• At home, n (%)	70 (91)^c^
with support from relatives, n (%)	14 (18.2)
with support from nursing care, n (%)	17 (22.1)
• Assisted living, n (%)	3 (3.9)^c^
• Care home, n (%)	2 (2.6)^c^

(a) missing data *n* = 2, (b) missing data *n* = 8, (c) missing data *n* = 4

Delirium was primarily caused by operations (*n* = 38, 49.4%) or infections (*n* = 29, 37.7%); however, in 14 cases (18.2%) the cause remained unclear (multiple selection of causes was possible). Prior to delirium onset, six patients (7.8%) received low-dose antipsychotics (i.e., melperone, pipamperone, risperidone), twelve (15.6%) were on antidepressants, eleven (14.3%) used opioids, and four (5.2%) were treated with anticholinergics for overactive bladder. Delirium was categorized as hyperactive in 45 participants (58.4%), hypoactive in 6 (7.8%), mixed in 9 (11.7%), whereas in 17 (22.1%) it remained unclassified. The average duration of delirium was 8 days (range 1–27, SD 7.0).

Of the 77 participants, 66 (85.7%) recalled feeling confused during the episode of delirium. Most (*n* = 49, 63.6%) were able to provide detailed comments on their experiences of confusion. The majority (*n* = 52, 67.5%) of participants reported primarily feeling afraid or anxious. Visual hallucinations were reported in *n* = 46 (59.7%), and auditory hallucinations in *n* = 17 (22.1%). More than half (*n* = 45, 58.4%) of the patients stated, that they were still affected by the experience. Fear of a new episode was reported by *n* = 33 (42.9%). In contrast, few (*n* = 5, 6.5%) participants stated that pain was either not noticed by the medical staff and/or not adequately treated. One-third of the patients recalled remembering certain moments as bizarre or even funny.

Patients’ subjective perception of the duration of delirium differs significantly in some cases from the duration recorded by staff. The majority of patients remembered the duration of delirium to be shorter (*n* = 34, 44.2%) than reported in the chart, only three (3.9%) perceived it to be longer, whereas *n* = 15 (19.5%) could not estimate the duration of delirium at all.

### Patients narrative on their subjective experience of delirium

In the following section, we present the most remarkable experiences of the three relevant themes identified from the qualitative content analysis.

1. Emotions during delirium.

Participants reported their experiences with fear and/or anxiety as “indescribable”, “dreadful”, “terrifying”, “extreme” and “inhuman”. One participant compared it to a “life-threatening feeling”. In some cases, fear was directed at specific topics, such as fear of being left alone or poisoned, or of losing personal belongings.

Less often than anxiety and/or fear, participants described feelings of anger. Particularly in situations where they were forced to stay in a room or bed, although they wanted to leave. Some participants reported that they felt insufficiently supported by medical personnel and relatives. Some of them reported having the impression that people were acting against them.

Among the three patients for whom physical restraints were used, “massive fear and anger” was felt. They reported feelings of being locked and/or trapped. “Experiencing the situation and not getting out of it” and “begging to be untied again”. One participant reported feeling that “nobody cared for him” while he was physically restrained and he was afraid of being punished and sent to jail due to his disobedience.

2. Hallucinations and dream-like experiences.

Some participants (*n* = 27/77) were able to give detailed descriptions of their primarily visual hallucinations. Participants reported that, “Building blocks were flying in my direction.”, “Shadows were behind me.”, “Changing colours were on the ceiling.”, and “Foreign people were in my room.” Auditory hallucinations were reported as “noises fading away (from afar) and growing louder (swell)”, or as “a loud bang”. Tactile hallucinations were also experienced. For example, patients mentioned a “feeling of floating”, as well as the “feeling of being turned around again and again”.

Most often participants remembered dream-like experiences, which often had a link to reality or sometimes the content was associated with the patient`s biography. Examples are: “I didn`t want to go to the hospital because I thought they were going to extract my kidney.”, “The hospital was only a fake hospital. In reality, the medical personnel were murderers.”, “I was supposed to be transferred to rehabilitation in the North of Italy, and thought about how to manage the transfer back home.”, or “I could see people belonging to a sect with a bed sheet. They wanted to catch me with this sheet, I should pee into it.”. Other examples include, “The room wasn`t a hospital room, and doctors were not actually doctors. I didn`t want to take pills from them because they were foreigners, but not doctors”. “A very tall doctor appeared at the side of my bed and dissolved into a ghost.”. “I was sure to be in the death chamber and that nobody wanted to tell me about.”, “It was raining so hard, the water ran down the walls, a very large tadpole came with the water into the room and ate the plants in the flower bed.”, “The room was full of calculation tasks, I was very busy solving these tasks.”, and “I was with a friend in a tent, furniture was sold there.”.

3. Changes of feelings and memories over time/ coping

Fear of experiencing another delirium, fear of losing one’s mind, and fear of losing control were most often reported. Shame was also a recurring theme reported by four of the participants. One patient even went to the intensive care unit afterwards to apologize.

Another subset of participants (*n* = 12) commented that receiving orientation cues was helpful, such as the date on the printed menu on the meal tray, or personnel reassuring them which hospital they were in. Getting out of bed regularly and having a structure to the day were also considered beneficial. Conversations with family member or staff were also mentioned as being an aid in overcoming delirium. Especially receiving reassurance through conversations were mentioned as well as calming talks. Two participants reported having a positive memory of physical contact. Eight participants described that relatives, and ten that healthcare staff were very important in overcoming delirium.

During the initial contact many individuals (*n* = 21) reported that they were still affected by memories of the delirium and found them difficult to process and cope with. Conversely, the same number of participants stated that they had “overcome” their experience with delirium.

In the subgroup of *n* = 29 followed up at 3 months, *n* = 10 were still distressed by the experience of delirium and/or feared a future episode. The unpleasant memories and feelings were still very clear and vivid. Participants reported, “I was strapped, it was the worst thing that ever happened to me.”, “I still have the whole experience in front of me, it was horrible.”, and “I still feel sorry for the doctors, I am not usually like this”.

## Discussion

We obtained semi-structured interviews with 77 geriatric inpatients who had recovered from acute delirium. Fear and/or anxiety were the dominant feelings associated with the episode of delirium. Delirium was experienced as a highly stressful event. Shame and fear of a repeat episode can burden patients for at least three months.

An interview study of 15 patients who had suffered delirium during hip surgery reported similar findings. In this study delirium was often experienced subjectively as a sudden change in reality, which led to strong emotions such as fear, panic and anger. In some cases, the experiences were linked to unfulfilled physiological needs [10]. The disorientation in a world between reality and a dream, that was often mentioned by our patients, has also been repeatedly described in previous work [11]. Along with perceived limitations in cognition, feelings of isolation and a loss of control also arose in our participants and have been described in the literature [12].

The information provided by the few patients (*n* = 3) who remembered being immobilized/restrained shows how negatively this experience is perceived. Patients often perceived these restrictions as punishment and the situation was associated with loneliness and helplessness. Indeed, restraints in geriatrics are associated with numerous poor outcome parameters. In a retrospective study, Chou et al. [13] showed poorer functional status, a longer hospital stay and higher mortality in association with restraints. Okumura et al. came to similar conclusions in patients with dementia and pneumonia [14]. The negative memories of restraints in our study provide an additional argument against any form of restraint.

Consistent with our findings with a mean length of delirium of 8 days (SD 7.0), the average duration of delirium in hospitalized geriatric patients was reported to be 8 days with similar findings in older hospitalized patients (7 days (SD 9.1)) [15, 16]. The average duration of delirium in nursing homes was reported to be 11.3 days [17]. To the best of our knowledge, the discrepancy between the shorter estimated length of delirium by patients and the probable length documented in the medical record has not yet been investigated in other studies. The extent to which this reflects the limitation of cognitive abilities during delirium or possibly also mechanisms of psychological processing remains unclear.

With regard to helpful interventions, contact with staff and relatives were remembered as being particularly helpful. Specifically, recurrent reminders about the current location and situation (e.g., today’s date, name of hospital) during delirium. This confirms from the patients’ subjective point of view the importance of the recommendations to apply re-orienting communication at all times as highlighted in the NICE guidelines [18]. For some patients, touch was also perceived as being beneficial. Encouragement from relatives, and conversations about memories from the period of delirium were also often noted as positive. A limitation of this finding is that approximately one-third of the participants could not recall the delirium, and nine subjects did not recall the staff as being helpful during their dream-like experiences.

Above all, our study emphasizes the urgent need to act correctly and helpfully toward patients in the event of delirium. Guidance on this exists from e.g. the American Geriatric Society, the Society of Critical Care Medicine and the American Society of Anaesthesiologists [19–21] based on data from observational and interventional studies. The subjective experiences of our study participants underscore the recommendations to (a) avoid restraints, (b) introduce familiar and reorientating objects, (c) encourage the presence of family, (d) provide care through verbal communication and touch, (e) address possible feelings of shame and fear before discharge. Other recommendations were not reflected in our interview data, such as pain management, orientation aids, adequate fluid and food intake. However, it should be considered that all our participants were treated in geriatric settings in which delirium awareness is mandatory and part of education of the staff.

### Strengths and limitations

The study is the largest of its kind and includes quantitative and qualitative data. A small group (*n* = 29) received a 3-month follow-up. One limitation is that the comments were written down by the interview partner, with the possibility of an unintentional subjective influence. In most of the cases, the diagnosis of delirium was made by clinical diagnosis and not by a standardized instrument; however, all the study physicians were experienced geriatricians or neurologists. The end of delirium was always attested by a physician and verified by a standardized delirium screening tool. Due to the exclusion of participants with severe dementia and/or psychotic illnesses, our data may not comprehensively measure all aspects of delirium and does not reflect the experiences of all individuals affected by delirium, particularly those with dementia who are at highest risk for delirium. Given the inherent limitations of data collection with individuals with (advanced) dementia, this represents an important avenue for future work.

## Conclusions

Most patients overcoming delirium recalled unpleasant emotions, mainly anxiety and/or fear. After recovering from delirium, shame regarding the episode and fear of cognitive impairment were reported most often. Reassuring conversations, including re-orientation assistance, as well as touch were particularly helpful during delirium. This underscores the important role of healthcare professionals and relatives both during delirium and in the period following the episode, when patients are coping with its sequelae. Professionals working with geriatric patients are encouraged to implement the current guideline recommendations on treating delirium to help reduce the (lasting) negative (psychological) outcomes associated with delirium. Concerning further research, qualitative studies appear to be particularly necessary as they allow collection of more in-depth information about the patients lived experiences with delirium, including memories and associated emotions. role that 

## Supplementary Information

Supplementary Material 1.

Supplementary Material 2.

## Data Availability

The data that support the findings of this study are available on reasonable request from the corresponding author.

## Abbreviations

Univ. Ol: Geriatric Medicine, Carl von Ossietzky University Oldenburg
Hosp. Bhv: Department of Geriatrics, General Hospital Bremerhaven Reinkenheide gGmbH
Hosp. B: Department of Geriatrics, Agaplesion Bethesda Krankenhaus Bergedorf GmbH
Hosp. L: Department of Geriatrics, General Hospital Lüneburg GmbH
MMSE: Mini Mental Status Examination
4AT: Rapid Clinical Test for Delirium
CAM: Confusion Assessment Method
CAM-ICU: Confusion Assessment Method-Intensive Care Unit
