# Supplementary material for: The subjective experiences of patients recovering from delirium in acute geriatric care: An analysis of quantitative and qualitative interview data
Source: BMC Geriatr. 2026 Jan 23;26:265. doi: 10.1186/s12877-026-06992-z (PMC12930763; doi:10.1186/s12877-026-06992-z)
Supplement: Supplementary file 1 — Supplementary Material 1. [file 12877_2026_6992_MOESM1_ESM.docx]

Interview guide (English version)

| 1. Do you feel that you have been confused recently? Do you remember that?  - Yes - No | Comments |
| --- | --- |
| 1. Do you have the feeling that your mind is completely clear again?  - Yes - No | Comments |
| 1. Was this experience associated with fear?  - Yes - No | Comments |
| 1. Were there any strange moments during this time?  - Yes - No | Comments |
| 1. During this time did you experience anything unusual, for example...  - seeing something unusual (visual hallucinations)? - hearing something unusual? (acoustic hallucinations) - feeling something unusual (tactile hallucinations)? - smelling or tasting something unusual (olfactory or gustatory hallucinations)? - Further unusual experiences (not mentioned above) | Comments |
| 1. Are you still burdened by the experience or can you put it behind you? Do you still think about this experience?  - Yes - No | Comments |
| 1. Did you perceive the content of this experience as being part of your own self or personality?  - Yes - No | Comments |
| 1. What do you remember most?   8a. Were there any environmental factors or staff behaviors that were particularly helpful? If so, which ones?  8b. What was particularly unpleasant?  8c. Did you experienced pain, which was not noticed (by the staff)? | Comments |
| 1. Can you estimate the duration of your state of confusion?   The state lasted for:   - Less than an hour - A few hours - About 24 hours - Several days - I can`t estimate the duration | Comments |
| 1. Do you fear a further episode of delirium? | Comments |
| 1. Have you experienced a similar state before? | Comments |
