# Supplementary material for: The subjective experiences of patients recovering from delirium in acute geriatric care: An analysis of quantitative and qualitative interview data
Source: BMC Geriatr. 2026 Jan 23;26:265. doi: 10.1186/s12877-026-06992-z (PMC12930763; doi:10.1186/s12877-026-06992-z)
Supplement: Supplementary file 2 — Supplementary Material 2. [file 12877_2026_6992_MOESM2_ESM.docx]

Additional file 2. Information on implemented delirium prevention-bundles

| Delirium prevention-bundle in the geriatric department | Univ. Ol | Hosp. B | Hosp. Bhv | Hosp. L |
| --- | --- | --- | --- | --- |
| Educational training of staff | physicians, occupational and physiotherapists,  nurses: In-house training courses | partial (nurses, physicians) | occasional lectures | geriatric team (physicians, occupational and physiotherapists, nursing staff), online training for nursing staff through the facility |
| Measures to optimize the clinical/preoperative situation, including medication checks | + | + | + | + |
| Nutrition/fluid intake according to needs | + | + | + | + |
| Environmental anti-delirium measures (e.g., calendars, personal items, lighting) | + | calendar, clock | + | calendar, clock, personal items, automatic lighting to structure the day in the special dementia/delirium ward |
| Compensation for sensory impairments (glasses, hearing aids) | + | + | + | + |
| Structuring of daily routines and focus on needs | as part of multi-professional work in early geriatric rehabilitation | as part of early geriatric rehabilitation | as part of early geriatric rehabilitation | as part of early geriatric rehabilitation (e.g., group activities) in collaboration with nursing/therapy/doctors |
| Promotion of healthy sleep | daytime activation | no special measures | daytime activation | daytime activation, sleep teas, sleep hygiene advice |
| Reorientation measures/ activities | clocks/calendars; Orientation communication by geriatric care staff | none | none | clocks/calendars throughout geriatric care,  Orientation communication,  Color/light/room design in the special ward |
| Daily pain assessment and monitoring | + | + | + | + |
| Involvement of relatives, rooming in | + including rooming in in some cases | + including rooming in | + rarely rooming in | + including rooming in |
